# Management of adult patients with podocytopathies: an update from the ERA Immunonephrology Working Group

**DOI:** 10.1093/ndt/gfae025

**Published:** 2024-02-10

**Authors:** Safak Mirioglu, Lisa Daniel-Fischer, Ilay Berke, Syed Hasan Ahmad, Ingeborg M Bajema, Annette Bruchfeld, Gema M Fernandez-Juarez, Jürgen Floege, Eleni Frangou, Dimitrios Goumenos, Megan Griffith, Sarah M Moran, Cees van Kooten, Stefanie Steiger, Kate I Stevens, Kultigin Turkmen, Lisa C Willcocks, Andreas Kronbichler

**Affiliations:** Division of Nephrology, Bezmialem Vakif University School of Medicine, Istanbul, Turkey; Department of Immunology, Aziz Sancar Institute of Experimental Medicine, Istanbul University, Istanbul, Turkey; Division of Pediatric Nephrology and Gastroenterology, Department of Pediatrics and Adolescent Medicine, Comprehensive Center for Pediatrics, Medical University of Vienna, Vienna, Austria; Division of Nephrology, Marmara University School of Medicine, Istanbul, Turkey; Department of Renal Medicine, Addenbrooke's Hospital, Cambridge University Hospitals, Cambridge, UK; Department of Pathology and Medical Biology, University of Groningen, University Medical Center Groningen, The Netherlands; Department of Health, Medicine and Caring Sciences, Linköping University, Linköping, Sweden; Department of Renal Medicine, Karolinska University Hospital and CLINTEC Karolinska Institutet, Stockholm, Sweden; Department of Nephrology, Hospital Unversitatio Fundacion Alcorcon, Alcorcon, Spain; Division of Nephrology, RWTH Aachen University Hospital, Aachen, Germany; Department of Nephrology, Limassol General Hospital, Limassol, Cyprus; University of Nicosia Medical School, Nicosia, Cyprus; Department of Nephrology and Renal Transplantation, Patras University Hospital, Patras, Greece; Imperial College Healthcare NHS Trust Renal and Transplant Centre, Hammersmith Hospital, London, United Kingdom; Cork University Hospital, University College Cork, Cork, Ireland; Division of Nephrology and Transplant Medicine, Department of Medicine, Leiden University Medical Center, Leiden, The Netherlands; Division of Nephrology, Department of Internal Medicine IV, Hospital of the Ludwig-Maximilians-University, Munich, Germany; Glasgow Renal and Transplant Unit, Queen Elizabeth University Hospital, Glasgow, UK; Division of Nephrology, Department of Internal Medicine, Necmettin Erbakan University, Konya, Turkey; Department of Renal Medicine, Addenbrooke's Hospital, Cambridge University Hospitals, Cambridge, UK; Department of Internal Medicine IV, Nephrology and Hypertension, Medical University Innsbruck, Innsbruck, Austria

**Keywords:** FSGS, guideline, KDIGO, MCD, podocytopathy

## Abstract

The histopathological lesions, minimal change disease (MCD) and focal segmental glomerulosclerosis (FSGS) are entities without immune complex deposits which can cause podocyte injury, thus are frequently grouped under the umbrella of podocytopathies. Whether MCD and FSGS may represent a spectrum of the same disease remains a matter of conjecture. Both frequently require repeated high-dose glucocorticoid therapy with alternative immunosuppressive treatments reserved for relapsing or resistant cases and response rates are variable. There is an unmet need to identify patients who should receive immunosuppressive therapies as opposed to those who would benefit from supportive strategies. Therapeutic trials focusing on MCD are scarce, and the evidence used for the 2021 Kidney Disease: Improving Global Outcomes (KDIGO) guideline for the management of glomerular diseases largely stems from observational and pediatric trials. In FSGS, the differentiation between primary forms and those with underlying genetic variants or secondary forms further complicates trial design. This article provides a perspective of the Immunonephrology Working Group (IWG) of the European Renal Association (ERA) and discusses the KDIGO 2021 Clinical Practice Guideline for the Management of Glomerular Diseases focusing on the management of MCD and primary forms of FSGS in the context of recently published evidence, with a special emphasis on the role of rituximab, cyclophosphamide, supportive treatment options and ongoing clinical trials in the field.

## INTRODUCTION

The pathogenesis that triggers minimal change disease (MCD) and focal segmental glomerulosclerosis (FSGS) is complex. Genetic variants, epigenetic, immunological, metabolic, and viral factors may contribute to disease development. MCD and FSGS are historically defined histological entities complemented by clinical criteria, particularly the response to immunosuppressive therapy. Yet, the observed histopathological lesions do not align well with their diverse underlying pathologies and it remains possible that MCD and FSGS represent a disease continuum with a common pathogenesis rather than two separate disease entities [1]. The uncertainty concerning the pathogenesis of MCD and FSGS can preclude optimal management. Moreover, most studies focusing on adults included patients with one histological lesion, rather than studying MCD and FSGS together. Therefore, we will evaluate them separately.

MCD is the most common pediatric glomerulopathy and accounts for 10–15% of nephrotic syndrome in adults, while FSGS is primarily seen in adults. Primary forms of adult MCD and FSGS present with a similar extent of proteinuria, but often exhibit a different disease course with immediate steroid response in MCD and a slower/absent response in FSGS. Activation of protease activated receptor 1 (PAR-1) [2], a podocyte membrane protein, has been suggested as a key initiator of the presumed circulating soluble factor, responsible to initiate FSGS. There is emerging evidence that a subset of patients with MCD have autoantibodies against podocyte proteins (for example, nephrin), providing potential links between podocyte injury, autoimmunity, and proteinuria response to anti-B-cell treatment [3]. Recently, anti-nephrin antibodies have been identified in 11 (79%) patients with recurrence of primary FSGS after kidney transplantation [4], further adding evidence to the theory of a continuous disease spectrum of MCD and FSGS, and that pathobiology needs to be re-written based on antibody detection.

These clinical observations suggest a heterogeneous pathogenesis underlying the current disease classification. As a result, the enrollment of patient populations with divergent causes of disease may have contributed to the failure of several clinical trials, and personalized treatments for MCD and FSGS are currently unavailable. Enhanced phenotyping may become possible through the emergence of novel biomarkers from well-defined cohorts, such as North American Nephrotic Syndrome Study Network (NEPTUNE), as these allow for the identification of relevant disease pathways. For example, the detection of tumor necrosis factor (TNF) kidney pathway activation by molecular profiling identified a subgroup of patients with either MCD or FSGS. This discovery led to the design of a clinical trial testing TNF inhibition in a subgroup with a greater likelihood of disease progression [5].

This article considers the evidence supporting the KDIGO 2021 Clinical Practice Guidelines for the Management of Glomerular Diseases (KDIGO 2021 GD) [6], and highlights published and upcoming developments that might influence adjustments of guidelines and impact treatment decisions with particular considerations regarding immunosuppressive therapies, podocyte-directed drugs, or agents used to optimize chronic kidney disease (CKD) management. Few major advances have been reported since the publication of KDIGO 2021 GD; however, this article will dissect certain KDIGO recommendations and will also highlight the need for an upgrade of rituximab, which seems to be particularly effective in managing cases with MCD. Additional work can be found in the supplementary data (see online supplementary material), as we focus on alternative approaches when patients have relapsing/resistant disease, calcineurin inhibitor therapy in FSGS, extracorporeal therapies and anticoagulation with a specific focus on MCD/FSGS.

### Diagnosing MCD and FSGS

**Figure 1:**
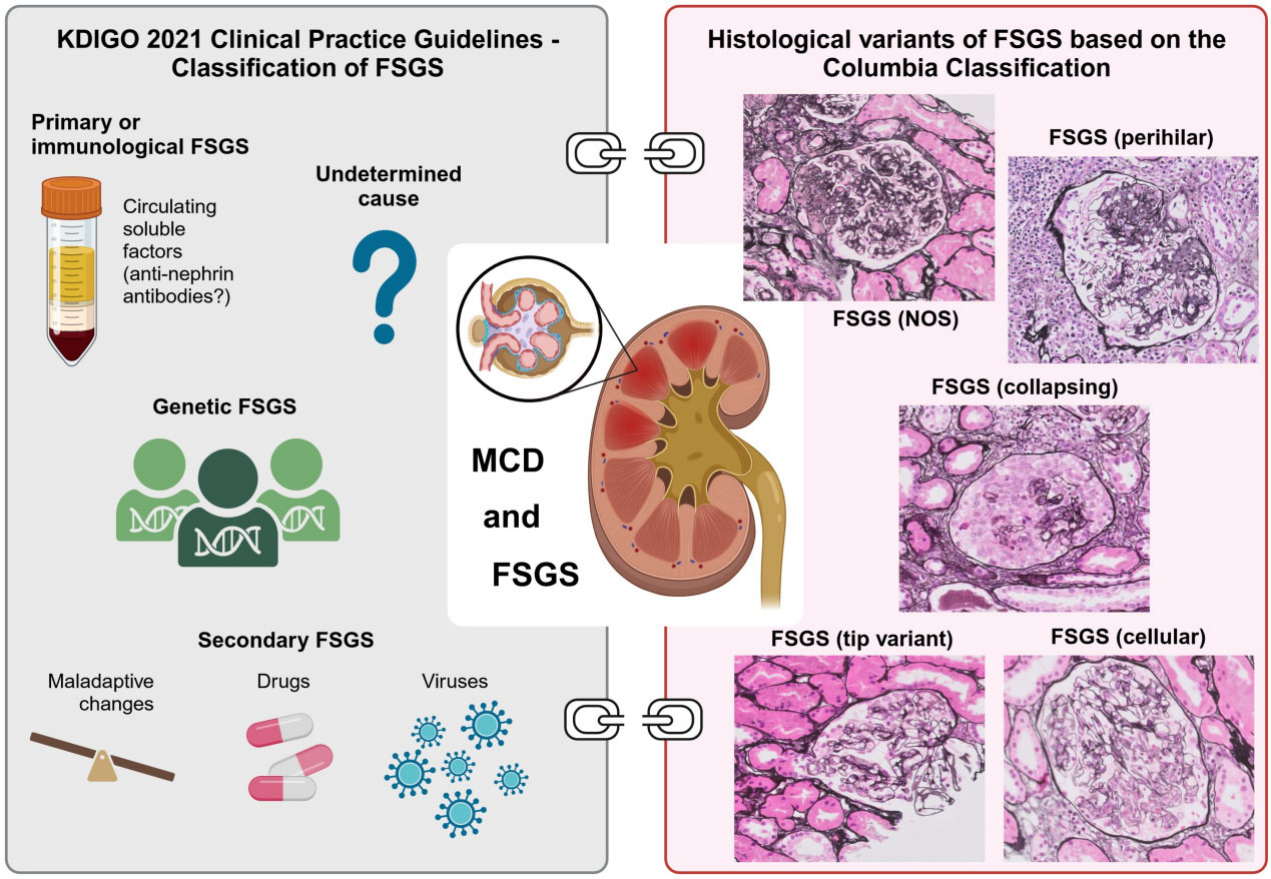
The KDIGO 2021 Clinical Practice Guidelines (left) highlight that a correct classification is pivotal to choose the correct management strategy. Immunosuppression might only be started in patients with a primary FSGS form, which is likely due to circulating factor(s). Emerging new variants contribute to the large group of genetic forms, and of course different stimuli can induce a secondary form of FSGS. The Columbia Classification (right) proposed the use of five different histological patterns. These patterns do not correlate with treatment response in ‘presumed’ primary cases, nor do they allow to distinguish between primary and secondary forms of FSGS. FSGS: focal segmental glomerulosclerosis; MCD: minimal change disease; NOS: not otherwise specified. Parts of the figure were created with BioRender.com.

Histopathology and/or histologic lesions underlie a diagnosis of MCD or FSGS subtypes. In MCD, light microscopy shows no glomerular lesions or only mild focal mesangial prominence. Immunofluorescence is negative or shows low-intensity mesangial staining for IgM, which is sometimes accompanied by C3 or C1q [7]. Extensive foot process effacement is the hallmark finding of MCD on electron microscopy, and podocyte injury might also entail presence of cytoplasmic vacuoles and microvillus transformation [8]. FSGS is a histologic pattern and does not allow differentiation between primary forms, genetic variants nor secondary forms due to alterations of glomerular epithelial cells or adaptive changes with glomerular hypertension. The eponymous feature of scarring/sclerosis is seen by light microscopy. The Columbia classification subdivides FSGS into five different lesions. These lesions are neither pathognomonic nor do they allow differentiation between different FSGS etiologies (Fig. 1). Repeat kidney biopsy per indication (worsening kidney function, persistent proteinuria, and relapse as most common reasons) found a subtype change in 11 (46%) patients, and a majority (82%) transitioned to a not otherwise specified (NOS) pattern [9], further underlining that different histopathological lesions are present in FSGS and are not helpful to predict activity nor choice of therapy.

Typical FSGS lesions can be observed in biopsies when the limits of the compensatory processes to glomerular hyperfiltration or injury have been exceeded, and it is often perceived as a sign of maladaptive changes rather than primary FSGS [10, 11]. Immunofluorescence might reveal non-specific IgM or C3 staining in sclerotic areas, which in general co-localize and points toward complement activation by IgM [12]. On electron microscopy, the extent of foot process effacement (FPE) was found to be helpful for differentiating primary and maladaptive FSGS but not in detecting genetic forms of FSGS [13]. A recent study found that all patients with a primary form had FPE of more than 80%, while no patients with underlying genetic variants or maladaptive forms had FPE of more than 50% [14]. However, cases of presumed primary FSGS with lower FPE have been reported [6]. The extent of FPE might aid differentiation in a suspected case of primary FSGS but reliance on histology alone is not warranted. When FSGS cannot be classified by pathological assessment, genetic analysis via next generation DNA sequencing should be offered to patients [15]. Thus, a careful evaluation to stratify patients with FSGS is needed for treatment decisions to improve therapeutic outcomes, which we discuss in detail below.

### Treatment of MCD and FSGS: general considerations

Advancing CKD is unusual in patients with steroid-sensitive MCD, while acute kidney injury can be seen in the context of high-grade proteinuria and hypoalbuminemia [16]. Initial response to glucocorticoids is critical and informs prognosis because steroid-resistant disease is the strongest independent predictor of kidney failure [17]. Steroid resistance is seen in 8–25% of patients in various series although true prevalence is likely lower in MCD compared with FSGS. Typically, FSGS lesions are encountered in repeat biopsies of such patients [16, 18]. High-grade proteinuria, impaired kidney function, FSGS lesions on biopsy, degree of interstitial fibrosis and tubular atrophy on the specimen all predict risk of progression to end-stage kidney disease (ESKD) [17].

In the KDIGO 2021 GD guideline [6], four diagnostic groups for FSGS were suggested: primary, genetic, secondary, and FSGS of undetermined cause (FSGS-UC). This classification is of importance in defining patients likely benefitting from immunosuppressive treatment. In addition to FSGS due to genetic and known secondary causes, the KDIGO Work Group suggested that patients with FSGS-UC who present without nephrotic syndrome should not be treated with immunosuppressive agents. Instead they should be monitored for an increase in proteinuria or development of nephrotic syndrome [6]. There is consensus that such cases might benefit from supportive measures but these would not respond to immunosuppressive agents [15].

The degree of proteinuria and initial response to treatment provide prognostic information in primary FSGS [17]. Over half of the patients with nephrotic-range proteinuria progress to ESKD, whereas those suffering from non-nephrotic proteinuria, which is more suggestive of secondary disease, have good outcomes with a 10-year kidney survival rate of 90% [6, 19]. Partial remission heralds a better prognosis with a 75% kidney survival at 10 years [19, 20].

MCD is known for its relapsing nature. Approximately two-thirds of patients may suffer from at least one episode of relapse after remission while up to one-third becomes frequently relapsing (FR) or steroid-dependent (SD) [21]. Compared to the high response rate of MCD with more than 80% achieving remission with steroids, FSGS has lower response and relapse rates: 47–66% of patients remit, 25–36% of whom relapse [19, 22]. Steroid resistance (SR) is seen in 10–20% of patients with MCD. There is an ongoing debate as to whether these patients instead have FSGS: repeated biopsies may show FSGS lesions given the focal nature of the disease [21, 23]. SR is a significant problem in FSGS, which is encountered in 40–60% of patients [23, 24]. Because SR is found in all forms of genetic FSGS, there is a broad agreement that genetic testing is likely beneficial in this selected patient group, particularly if presenting in adolescence or earlier adulthood [6]. Underlying genetic causes of FSGS should be considered when resistance to glucocorticoids and other immunosuppressive measures, familial history of kidney disease and/or parental consanguinity, presence of extrarenal features of inherited traits, and evidence of early progression to CKD are present [25]. Among the genetic causes, we believe two of them require special attention: *APOL1* and *COL4A*. Variants of the *APOL1* gene conferred an increased risk as high as 17-fold for the development of FSGS, and were associated with earlier age of onset and a faster progression to ESKD [26]. The risk is quite high in individuals with two risk variants, but the ones with only one risk variant can still suffer from kidney disease after a ‘second hit’, which is generally a chronic viral infection, especially human immunodeficiency virus [27]. FSGS associated with collagen disorders due to variants of *COL4A3, COL4A4*, and *COL4A5* are usually underdiagnosed because a considerable amount of the patients with these pathogenic variants do not have clinical manifestations of Alport syndrome or thin basement membrane diseases [28].

Cyclosporine has been reported to be useful in some patients with genetic FSGS [29], possibly due to its effects on glomerular hemodynamics and podocyte cytoskeleton. A podocyte-stabilizing effect was also proposed for rituximab, as it prevented downregulation of sphingomyelin phosphodiesterase acid-like 3b protein and its acid sphingomyelinase activity by recurrent FSGS sera, thereby reducing the apoptosis of podocytes [30]. These findings substantiate the use of these agents as ‘podocyte’-protective therapies.

### Steroids as initial therapy in MCD and FSGS

**Figure 2:**
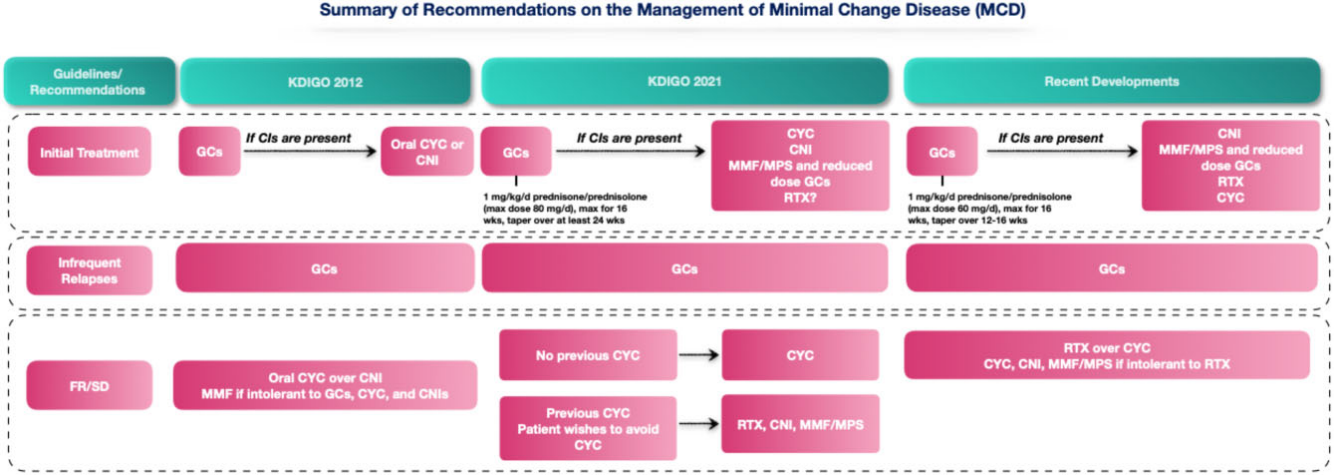
Summary of the recommendations on the management of minimal change disease in 2021 Kidney Disease: Improving Global Outcomes (KDIGO) Clinical Practice Guideline for the Management of Glomerular Diseases compared to the 2012 guideline [6, 70]. CI: contraindication; CNI: calcineurin inhibitor; CYC: cyclophosphamide; FR: frequently relapsing; GC: glucocorticoid; MCD: minimal change disease; MMF: mycophenolate mofetil; MPS: mycophenolate sodium; KDIGO: Kidney Disease: Improving Global Outcomes; RTX: rituximab; SD: steroid-dependent.

Glucocorticoids remain the mainstay of initial therapy for both entities (Table S1, see online supplementary material). As highlighted by the Cochrane Database of Systematic Reviews summarizing interventions used in the management of adult MCD, the evidence certainty for use of glucocorticoids to induce remission is very low [31]. The Collaborative Study of Glomerular Disease trial randomized patients in a 1:1 fashion to receive prednisone (average over 6 months >25 mg/d, with slow taper thereafter) or placebo, and found that proteinuria was reduced more rapidly in the active therapy arm. Four patients (28.6%) in the control arm had a doubling of serum creatinine and three were initiated on glucocorticoids, leading to complete remission [32]. The KDIGO 2021 GD guideline acknowledges the lack of randomized clinical trial (RCT) evidence, but recommends high-dose glucocorticoids for a maximum duration of 16 weeks [6]. Tapering of glucocorticoids might be started two weeks after obtaining complete remission, but should be maintained for at least 24 weeks (Fig. 2). A recent RCT tested tacrolimus monotherapy *versus* prednisolone in adults with *de novo* MCD. In the MINTAC trial, patients allocated to the prednisolone arm received an initial prednisolone dose of 1 mg/kg/d (maximum 60 mg); after achieving complete remission, the dose was halved and maintained for 4–6 weeks before glucocorticoids were tapered over a further six weeks, ensuring that patients received a maximum of 16 weeks of glucocorticoid therapy. In the prednisolone arm, 84% and 92% achieved remission at 8 and 16 weeks of follow-up [33]. A MINTAC-based glucocorticoid therapy seems timely and is embedded in clinical practice recommendations in most centers. The TURING trial (Table 1; ISRCTN16948923) offers the treating physicians the choice between two different tapering regimens. The low-dose group has a reduction of cumulative glucocorticoids by 910 mg in comparison to the standard dose.

**Table 1:** Comparison of options of oral prednisone/prednisolone used in the management of MCD (and FSGS in some cases). Specific considerations were made for patients on long-term glucocorticoids and the inability to withdraw steroids in the TURING trial (ISRCTN16948923). Notably, TURING will also include patients with FSGS.

Topic	KDIGO 2021 [6]	MINTAC [33]	TURING high-dose	TURING low-dose
Initial dose	1 mg/kg/d (max. 80 mg/d) or 2 mg/kg every other day (max. 120 mg/d) for a minimum of 4 weeks, and a maximum of 16 weeks. Taper might be started 2 weeks after CR is obtained.	1 mg/kg/d (max. 60 mg/d); maintained for another week when CR is obtained	1 mg/kg/d (max. 60 mg/d) for up to 16 weeks; max. 2 weeks if PR/CR is obtained	1 mg/kg/d (max. 60 mg/d) for up to 16 weeks; max. 2 weeks if PR/CR is obtained
Tapering strategy	Tapering over at least 24 weeks	½ dose for another 4–6 weeks, then further tapering and withdrawal over 6 weeks	Thereafter: 40 mg (2 weeks), 30 mg (2 weeks), 25 mg (2 weeks), 20 mg (2 weeks), 15 mg (2 weeks), 10 mg (2 weeks), 5 mg (2 weeks), 2.5 mg (2 weeks)	Thereafter: 30 mg (2 weeks), 20 mg (2 weeks), 15 mg (2 weeks), 10 mg (2 weeks), 5 mg (2 weeks), 2.5 mg (2 weeks)
Time/cumulative dose	Tapering over at least 24 weeks	Tapering over 10–12 weeks (total treatment time at least 16 weeks)	Tapering over 16 weeks	Tapering over 12 weeks Reduction by 910 mg (in comparison to high-dose)

CR: complete remission; FSGS: focal segmental glomerulosclerosis; KDIGO: Kidney Disease: Improving Global Outcomes; MCD: minimal change disease; PR: partial remission.

**Figure 3:**
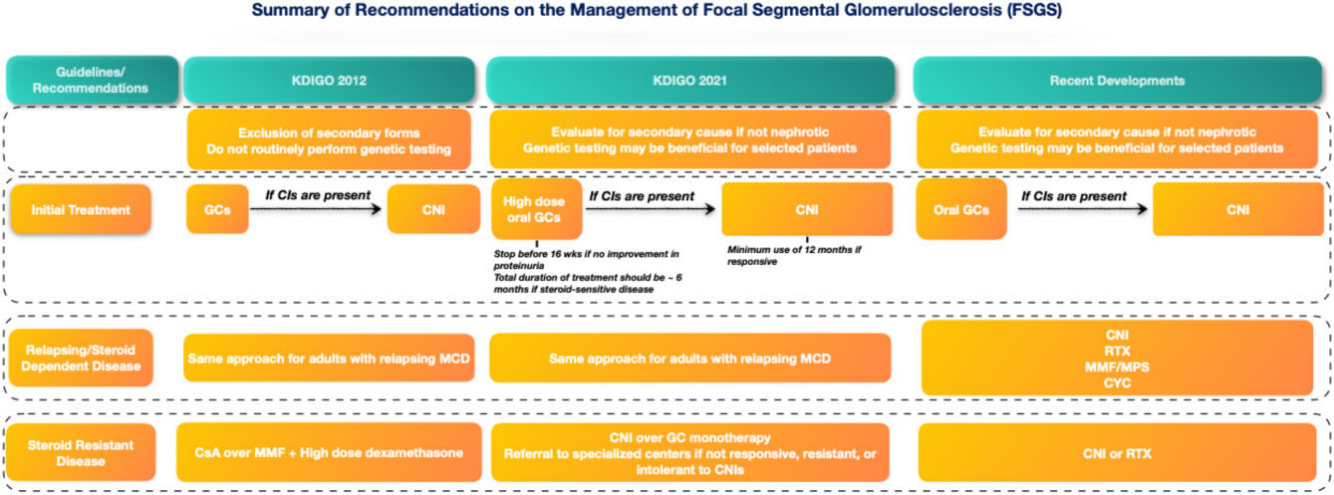
Summary of the recommendations on the management of focal segmental glomerulosclerosis in 2021 Kidney Disease: Improving Global Outcomes (KDIGO) Clinical Practice Guideline for the Management of Glomerular Diseases compared to the 2012 guideline [6, 70]. CI: contraindication; CNI: calcineurin inhibitor; CsA: cyclosporine; CYC: cyclophosphamide; FSGS: focal segmental glomerulosclerosis; GC: glucocorticoid; KDIGO: Kidney Disease: Improving Global Outcomes; MCD: minimal change disease; MMF: mycophenolate mofetil; MPS: mycophenolate sodium; RTX: rituximab.

No RCT comparing glucocorticoids to placebo in the management of FSGS has been conducted. The KDIGO 2021 GD guideline recommends an initial dose as used to manage MCD, and provides guidance on tapering (Fig. 3). These recommendations were mostly derived from trials including pediatric populations and observational studies [20, 23]. Dose and duration of treatment are also subject to the same limitations. The KDIGO 2021 GD guideline recommends high-dose glucocorticoids with 1 mg/kg prednisone daily (maximum 80 mg) or alternate-day dose of 2 mg/kg (maximum 120 mg); however, the evidence supporting alternate-day dosing is weak, originating from only one observational study [6, 34]. Importantly, a patient with no proteinuria response at 16 weeks is unlikely to benefit from continuing high-dose glucocorticoid therapy.

For both entities, further efforts need to be made to define ideal glucocorticoid duration in adults, in order to tailor glucocorticoid exposure to an individual's need and thus reduce side effect burden associated with long-term glucocorticoid use. Both entities have a relapsing/remitting nature, and most steroid-sensitive patients often need several courses of glucocorticoids during their disease course. Protracted use may cause harm, especially in patients with obesity, psychiatric diseases, poorly controlled diabetes, or osteoporosis. In these cases, other immunosuppressive agents can be introduced as discussed below, with a particular focus on the KDIGO 2021 GD guideline and potential change in order to initiate these agents.

### Rituximab: should it be ‘upgraded’?

Rituximab is currently recommended during relapsing courses of both entities if there is a previous use of cyclophosphamide or a patient wishes to avoid cyclophosphamide exposure.

The recent discovery of anti-nephrin antibodies as a driver of autoimmunity in a subset of MCD underlines the importance of B cells in the pathogenesis of MCD [3]. There is evidence that rituximab is effective in the management of adult MCD [35]. A retrospective comparison of different second line therapies used in a single center to manage FR/SD MCD indicated that the median time to relapse after rituximab was longer (66 *versus* 28 months) compared with other measures (CNIs, mycophenolate mofetil, and cyclophosphamide) but the difference fell short of statistical significance [36]. A recent meta-analysis of FR/SD MCD patients reported a complete remission rate in 87.3% of individuals receiving rituximab [37]. Case series of repeated rituximab administrations at 4- to 6-monthly intervals are emerging. One such study from Japan started such a regimen after one patient relapsed eight months after the first series of rituximab induction. The remaining 12 patients received rituximab 6-monthly and were maintained in remission during follow-up [38]. Rituximab in the management of MCD is currently tested in two randomized clinical trials, RIFIREINS (NCT03970577) and TURING (ISRCTN16948923). The RIFIRENS trial tests the use of rituximab *versus* continuous glucocorticoid administration in patients with steroid-induced remission of a first episode of MCD, while the TURING study includes either newly diagnosed or relapsing MCD or FSGS. A smaller RCT tested the potential of rituximab to maintain remission and focused on investigations of T-cell subsets. Nine of 10 patients remained in remission, and remission was associated with a significant decrease in the frequency of CD4^+^CD45RO^+^CXCR5^+^, invariant natural killer T cells (INKT) and CD4^−^CD8^−^ (double-negative, DN) T cells expressing the invariant Vα24 chain (DN-TCR Vα24) T cells [39], also highlighting that rituximab exerts effects on the immunological synapse, thereby exerting modulation of T cells.

The evidence for rituximab's efficacy in FSGS is more limited [40], originating mostly from small case series. In addition, the total number of patients included with (primary) FSGS has been restricted. Remission rates are lower in comparison to MCD [37]. The underlying pathology of FSGS might be related to secondary causes rather than primary disease in some case series where individuals with secondary FSGS likely were included, substantiated by limited FPE on electron microscopy in some cases, with a mean serum creatinine of 2.6 mg/dl at baseline and a mean age of 64 years [41]. The best current evidence stems from a multi-center study from Italy reporting on 31 patients with (primary) FSGS, of whom 18 (58%) had SD and 11 (35%) had SR disease. Response to therapy, defined as reduction of proteinuria to <3.5 g/d and at least to <50% from baseline was achieved in 45% and 43% at 6 and 12 months, when patients retreated with rituximab were included in the analysis. Independent predictors of response were steroid-dependence as indication and a proteinuria less than 5 g at baseline [42]. This again indicates that those not responding to glucocorticoids and to other immunosuppressive measures such as calcineurin inhibitors are less likely to achieve a therapeutic response to rituximab. The onset of nephrotic syndrome in such cases might be due to a genetic variant or is secondary to other triggers rather than having a primary underlying causative factor potentially amenable to immunosuppression. In addition, several studies, a majority conducted in a pediatric population, evaluated other predictors of response in patients who received rituximab (Table 2) [39, 43–49]. In a cohort of 22 consecutive patients with FSGS, T-cell activation markers were evaluated before administration of rituximab. Recovery of CD154^+^CD4^+^CD3^+^, interferon-gamma^+^CD3^+^, and IL2^+^CD3^+^ T cells 6 months after the treatment predicted the response [50]. In 102 children or young adults, higher levels of circulating levels of memory B cells at the time of rituximab administration were associated with relapse risk after therapy [46]. Proteinuria selectivity index (PSI), which is the ratio between the urinary clearance of IgG to transferrin is a predictor of steroid response in nephrotic syndrome, and a value of ≤0.20 has been related to higher remission rates. In a study of 29 adult patients (15 MCD and 14 FSGS) who were treated with rituximab, all patients with PSI ≤0.20 attained remission while no patients with PSI >0.20 did. Responses to previous glucocorticoids and other immunosuppressive agents were lower in the PSI >0.20 group, and most of these patients suffered from FSGS [51]. Soluble urokinase plasminogen activator receptor (suPAR) is one proposed circulating factor. A study focusing on patients with resistant FSGS and higher suPAR (>3500 pg/ml) levels did not find a benefit from rituximab administration [52], but it should be kept in mind that steroid-resistant patients, as mentioned above, are in general non-responsive to several lines of immunosuppression and unlikely have a primary FSGS form.

**Table 2:** Proposed predictors of relapse following rituximab in the management of minimal change disease and focal segmental glomerulosclerosis.

Study author	(Bio)marker under investigation	Main outcome	Population under investigation
Angeletti *et al*. [43]	Anti-rituximab antibodies	7/14 (50%) with and 28/40 without (70%) relapsed	Children with SDNS
Boumediene *et al.* [39]	CD4^+^CD45RO^+^CXCR5^+^, invariant natural killer T cells (INKT) and CD4^−^CD8^−^ (double-negative) T cells	Remission after rituximab was associated with a significant decrease in their expression	Children with FRNS/SDNS
Chan *et al*. [44]	Courses of rituximab	Median relapse-free period was shortest with first rituximab treatment (10 months)	Children with FRNS/SRNS or multidrug-dependent NS
Chan *et al*. [45]	Regulatory T cells, PMA-stimulated IL-2, IFN- γ	ROC-AUC of 0.99, 0.84 and 0.91 to predict sustained remission (lower levels)	Childhood-onset SDNS/SRNS
Colucci *et al*. [46]	Age, levels of memory B cells	Lower relapse risk at an age ≥10 years, higher relapse risk with higher levels of memory B cells at baseline	Children and adolescents with FRNS/SDNS
Colucci *et al*. [47]	IgM on the surface of T cells	Higher T cell count on IgM (>400 MFI; *n* = 13) was predictive of relapse in the first 12 months	Children with SDNS
Colucci *et al*. [48]	Switched memory B cells	Switched memory B cells (>0.067%; >1.65 cells/µl) as predictor of relapse	Children with FRNS/SDNS
Fribourg *et al*. [49]	Switched B-cell subsets	Breg, CSM resting, CSM activated, CSM CD27- and antibody-secreting B cells significantly higher in relapsing patients	Children with SDNS

AUC: area under the curve; Breg: regulatory B cells; CSM: cell surface marker; FRNS: frequently relapsing nephrotic syndrome; IFN: interferon; MFI: mean fluorescent intensity; NS: nephrotic syndrome; PMA: phorbol myristate acetate; ROC: receiver operating characteristic; SDNS: steroid-dependent nephrotic syndrome.

### The use of cyclophosphamide as second-line therapy in FR/SD MCD

According to the KDIGO 2021 GD guideline, the recommendation to use cyclophosphamide as second-line therapy in adult FR/SD MCD largely stems from experience in children. However, based on the efficacy and safety of rituximab in FR/SD MCD (see above), rituximab has, since publication of the KDIGO 2021 GD guideline, become a realistic substitute as second-line therapy in the management of MCD. Alternative agents to glucocorticoids are necessary to reduce glucocorticoid toxicity and to maintain remission in individuals with FR/SD MCD. The evidence for cyclophosphamide use in such a scenario in adults is limited to retrospective investigations, but reports consistently showed that remission was maintained more effectively after cyclophosphamide-induced remission and some patients remained relapse-free during follow-up [22, 53]. The recommendation of the KDIGO 2021 GD guideline was based on two retrospective observational studies, one summarizing experience of patients managed at the National Institute of Health (NIH) between 1990 and 2005 [16] and the other including patients treated at a single institution between 1950 and 1993 [18]. In the NIH case series, 20 patients received cyclophosphamide, while 43 were treated with CNIs and 14 with mycophenolate mofetil. Remission rates did not differ between the different agents (55%, 63% and 64%, respectively). No difference was observed in a small subset of SD (29%) or SR (39%) individuals [16]. Based on the toxicity of alkylating agents, it is not expected that future RCTs in MCD will include a cyclophosphamide-based therapy arm, and no such trial is currently ongoing.

### Agents to reduce proteinuria and eGFR decline

Renin angiotensin system (RAS) blockade with angiotensin-converting enzyme inhibitors or angiotensin receptor blockers is an established treatment strategy that reduces proteinuria and slow eGFR decline, as these agents decrease intraglomerular pressure, thereby reducing glomerular hyperfiltration [54]. Also, RAS blockers have anti-inflammatory and antifibrotic properties [55]. In addition to these conventional agents, there are now new drugs that can control non-immune progression of CKD.

Sparsentan, a dual antagonist of the angiotensin type 1 receptor and the endothelin type A receptor, was tested against conventional RAS blockade [56]. In the phase 2 DUET trial including 109 patients with FSGS, sparsentan resulted in a greater reduction in proteinuria compared to irbesartan (45% *versus* 19%), and partial remission was achieved in 28% and 9% of patients, respectively [56]. However, hypotension and edema associated with the drug may be of concern. Because proteinuria reduction has been associated with better outcomes, it has been proposed that sparsentan may have a therapeutic role in the management of FSGS [57]. More recently, results of the phase 3 DUPLEX trial (NCT03493685) have been reported. Sparsentan did not achieve the primary endpoint, which was defined as the eGFR slope over 108 weeks of treatment, with a between-group difference in the eGFR slope of 0.3 ml/min/1.73 m^2^ in favor of sparsentan (annual decline of −5.4 versus −5.7 ml/min/1.73 m^2^). However, it resulted in a 50.0% reduction in proteinuria as compared to 32.3% with irbesartan alone [58]. The full effects on eGFR might not be evident in a 2-year follow-up and a longer period might be required for a more reliable interpretation.

The use of sodium-glucose transporter-2 inhibitors (SGLT2i) has changed the standard of care concept in CKD. DAPA-CKD and EMPA-KIDNEY trials demonstrated the efficacy of SGLT2i in decreasing the progression of CKD with proteinuria in patients with and without diabetes [59, 60]. In a pre-specified analysis of the DAPA-CKD trial which included 254 patients with a biopsy-confirmed IgA nephropathy, SGLT2i use attenuated the risk of CKD progression [61]. Results were also similar for 104 patients with FSGS although it did not reach statistical significance [62]. In contrast, no such effect was reported in the EMPA-KIDNEY trial, and the overall effect of SGLT2i, when data were pooled, on kidney disease progression of FSGS was limited [63]. Notably, patients on immunosuppressive agents have been generally excluded from these trials and importantly, these disappointing results further argue that RCTs in cases of ‘FSGS’ (on a renal biopsy) do not make sense any longer. It should be kept in mind that both SGLT2i and sparsentan or using a combination therapy such as dapagliflozin and zibotentan, as has proven effective in the phase 2 ZENITH-CKD trial [64], can expand the CKD treatment armamentarium, and the efficacy of these agents in nephrotic syndrome should be evaluated separately and possibly in combination to identify any potential synergistic effects. In addition, the endothelin type A receptor antagonist atrasentan is currently being studied in a phase 2, open label trial (NCT04573920). Lastly, finerenone, a non-steroidal, selective mineralocorticoid receptor antagonist, has been shown to slow CKD progression once added to RAS blockers in patients with diabetic kidney disease [65]. Whether these effects will be replicated in MCD/FSGS or other primary glomerular diseases is not known yet, but there is an ongoing trial of finerenone in patients without diabetes and CKD (NCT05047263).

### Ongoing studies

**Figure 4:**
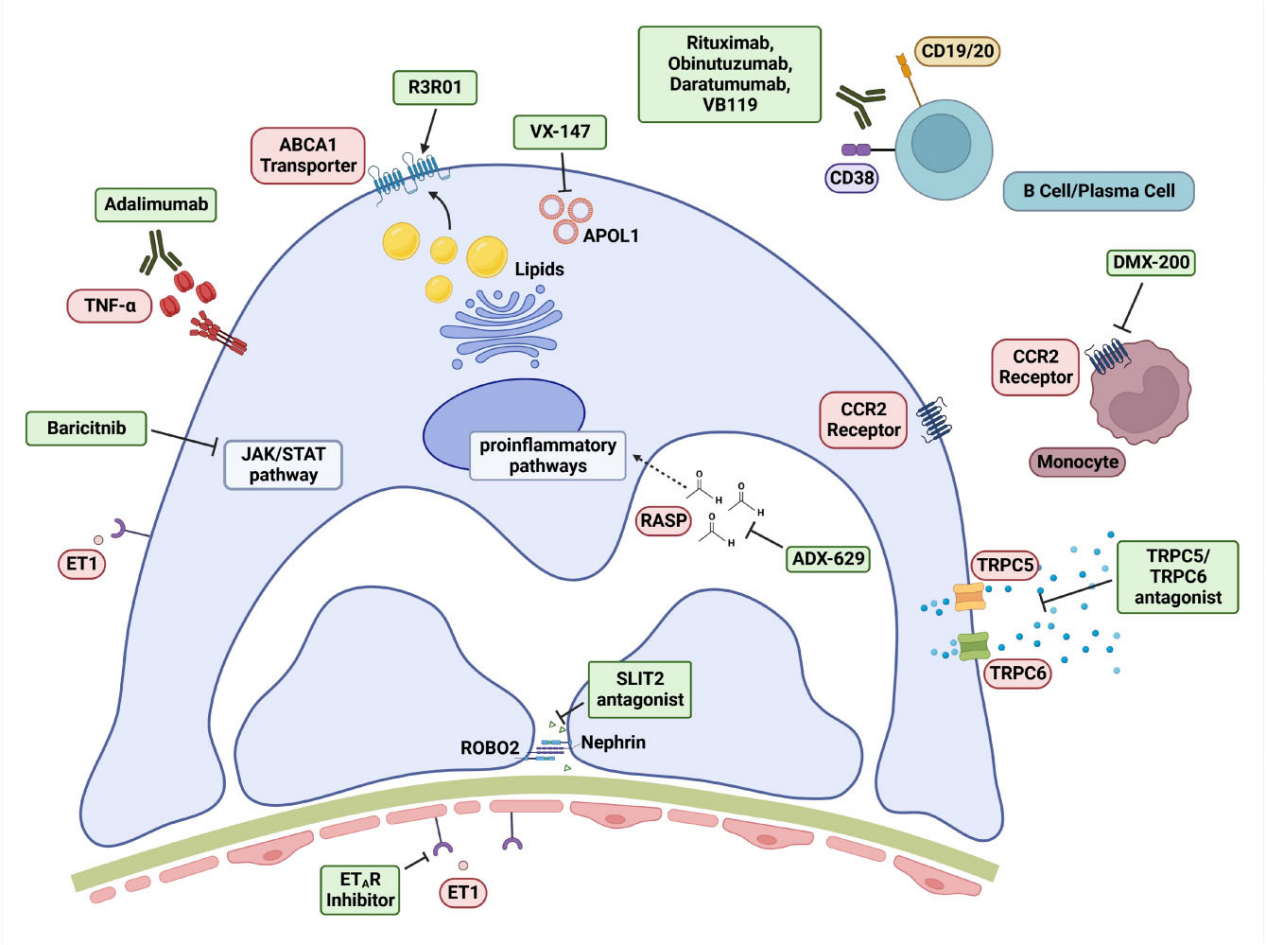
A schematic overview of potential drug targets, and emerging therapies tested or used in the management of podocytopathies. These potential candidates include immune system suppressing, immunomodulatory, podocyte skeleton stabilizing, and therapies aiming to lower systemic and intraglomerular hypertension. Modified from de Cos *et al*. [66] and created with BioRender.com.

Recently, trials on emerging therapies targeting different pathogenic signaling cascades in MCD and FSGS are evolving (see www.ClinicalTrials.gov for more information), including immunosuppressive acting agents (e.g. anti-CD20 monoclonal antibodies), causative directed therapies (e.g. APOL1 antagonists), podocyte specific therapies (e.g. TRPC5/6 channel inhibitors), and agents with antifibrotic/hemodynamic effect (e.g. endothelin antagonists) (Table 3) [66]. The effect of anti-CD20 monoclonal antibody (mAb) rituximab on the incidence of relapses in a steroid-responsive population is currently being investigated (NCT03970577). Two trials testing obinutuzumab, a second generation anti-CD20 mAb with enhanced B-cell depleting potential, in FSGS (NCT04983888) and SD or FR nephrotic syndrome (NCT05786768), are currently ongoing/planned. The Dual 1 trial sets out to test the efficacy of rituximab in combination with the plasma-cell targeting anti-CD38 mAB daratumumab in patients with multidrug dependent and resistant nephrotic syndrome (NCT05704400). The combination of anti-CD20 and anti-CD38 mAbs is speculated to control the development of autoreactive long-lived plasma cells in patients where B-cell depletion alone failed to provide sustained remission [67]. VB119, an anti-CD19 mAb is currently tested in subjects with steroid-sensitive MCD or FSGS (NCT05441826). In a phase 2 study, patients with FSGS and treatment-resistant MCD and increased urinary excretion of the biomarkers MCP1/Cr and/or TIMP1/Cr (markers for TNF-activation), are treated with anti-TNF-α mAb adalimumab (NCT04009668). ADX-629 is a novel, orally administered reactive aldehyde species (RASP) modulator. A phase 2 clinical trial in patients with MCD is planned with this agent (NCT05599815). A phase 2/3 study to evaluate the efficacy and safety of VX-147, a small-molecule inhibitor of APOL1, in FSGS and high-risk *APOL1* variants is ongoing (NCT05312879) after promising results from a phase 2a trial [68]. Moreover, JAK-STAT inhibition with baricitinib is investigated in *APOL1*-mediated podocytopathy (NCT05237388). TRPC5 and TRPC6, transient receptor potential cation channels in podocytes, contribute to intracellular calcium hemostasis. TRPC6 inhibition is currently studied in patients with FSGS (NCT05213624), whereas GFB-887, an inhibitor of TRPC5, was investigated as a potential therapeutic agent in FSGS and MCD (NCT04387448) in a phase 2 trial, which had to be terminated prematurely due to business reasons. R3R01, an investigational small molecule designed to decrease fat levels in kidney cells, is being studied in patients with primary FSGS (NCT05267262). ROBO2 expression is increased in glomerular disease. The ROBO2 fusion protein (PF-067301512) inhibits ROBO2/SLIT2 signaling but the study in FSGS has recently been terminated due to lack of efficacy (NCT03448692). In patients with FSGS, the phase 2 LUMINA-1 study evaluated CCX140-B, an orally administered selective antagonist of the C-C chemokine receptor type 2 (CCR2) (NCT 03536754). CCX140-B was not successful in reduction of proteinuria when compared to placebo. DMX-200 (repagermanium), another CCR2 inhibitor, designed to inhibit recruitment of monocytes implicated in inflammatory chemokine environment, is currently investigated in patients with FSGS and concomitant RAS inhibition therapy (NCT05183646). A schematic overview of potential drug targets is given in Fig. 4.

**Table 3:** A summary of ongoing trials in minimal change disease and focal segmental glomerulosclerosis, including the phase of the trial, inclusion of disease entity, and primary outcome measure.

NCT identifier	Compound	Pathway	Status	Phase	Inclusion criteria	Primary outcome measure
**NCT04983888**	Obinutuzumab	Anti-CD20 mAb	Recruiting	2	FSGS	Change^a^ in proteinuria in mg/24 h at months 6 and 12
**NCT04009668**	Adalimumab	Anti-TNF-α mAb	Recruiting	2	FSGS + TR-MCD	Change^a^ in MCP1/Cr and TIMP1/Cr level at week 10
**NCT03970577**	Rituximab	Anti-CD20 mAb	Recruiting	2	MCD	Incidence of relapse^b^ during 12 months following randomization
**NCT05786768**	Rituximab/Obinutuzumab	Anti-CD20 mAb	Not yet recruiting	2/3	MCD	Occurrence of relapse^c^ within 12 months following treatment initiation
**NCT05704400**	Rituximab + Daratumumab	Anti-CD20 mAb + anti-CD38 mAb	Not yet recruiting	2	MCD/FSGS	Safety: number of TEAEs; Efficacy: number of months in remission
**NCT05441826**	VB119	Anti-CD19 mAb	Active, not recruiting	2	MCD/FSGS	Proportion of subjects in remission at end of treatment; Incidence of SAEs, TEAEs, AESIs
**NCT05599815**	ADX-629	RASP modulator	Not yet recruiting	2	MCD/FSGS	Incidence and severity of TEAEs and SAEs
**NCT05213624**	BI 764198	TRPC6 inhibitor	Recruiting	2	FSGS	Number of patients achieving ≥25% reduction^a^ of UPCR at week 12
**NCT04387448**	GFB-887	TRPC5 inhibitor	Terminated due to business reasons	2	MCD/FSGS	Percent change^a^ in UPCR and UACR at week 12
**NCT03448692**	PF-06 730 512	Slit2 inhibitor	Terminated due to lack of efficacy	2	FSGS	Percent change^a^ in UPCR at week 13
**NCT05267262**	R3R01	Fat level reducer	Recruiting	2	FSGS	Incidence of AEs and change in UPCR at week 12
**NCT05312879**	VX-147	APOL1 inhibitor	Recruiting	2/3	FSGS (*APOL1* variants)	Percent change^a^ in UPCR at week 48; estimated eGFR slope at week 48; eGFR slope assessed at study completion
**NCT05237388**	Baricitinib	JAK-STAT inhibitor	Recruiting	2	FSGS (*APOL1* variants)	Percent change^a^ in UACR monthly for 6 months
**NCT01613118**	RE-021 (Sparsentan)	Endothelin A and angiotensin II receptor blocker	Active, not recruiting	2	FSGS	Percent change^a^ in UPCR at week 8
**NCT05003986**	RE-021 (Sparsentan)	Endothelin A and angiotensin II receptor blocker	Recruiting	2	MCD/FSGS	Incidence of TEAEs, SAEs, AEs leading to treatment discontinuation and AEIs; change^a^ in UPCR over 108 weeks
**NCT04573920**	Atrasentan	Endothelin-receptor antagonist	Recruiting	2	FSGS	Change^a^ in UPCR at weeks 12; change in UPCR at week 24
**NCT05183646**	DMX-200 (Repagermanium)	CCR2 inhibitor	Recruiting	3	FSGS	Change^a^ in UPCR at week 35; eGFR slope at week 35 and 104

AE: adverse events; AEI: adverse event of interest; FSGS: focal segmental glomerulosclerosis; mAb: monoclonal antibody; MCD: minimal change disease; SAE: serious adverse event; TEAE: treatment-emergent adverse event; TR-MCD: treatment resistant minimal change disease.

^a^change assessed in relation to baseline; ^b^defined as UPCR [urine protein to creatinine ratio of >2.65 g/g and decreased albumin level (<30 g/l)]; ^c^defined as UPCR of 2 g/g of creatinine or higher.

## CONCLUSION

MCD/FSGS in adults are a historically understudied area of nephrology, leaving the treating physicians with therapies used in the management of these lesions since the mid-twentieth century. However, MCD/FSGS are an attractive topic for both clinical and basic research, and represent an intriguing but also promising challenge for precision medicine. Recent interest from pharmaceutical companies and funding bodies has helped to delineate the understanding of disease pathogenesis in greater detail, and expand research for finding therapies with a good safety and efficacy profile.

The primary clinical focus in the management of MCD/FSGS must be prevention of CKD progression and reducing exposure of patients to immunosuppression, if nephrotic syndrome occurs as a secondary etiology. In primary cases, the reduction of proteinuria, ideally achieving proteinuria levels <1.5 g/g creatinine [69], has direct implications on long-term kidney prognosis. Uncertainty exists regarding which immunosuppressive therapies should be used and the duration, especially in reducing the risks associated with cumulative glucocorticoid exposure. However, recommendations are largely derived from experiences in children and observational studies, the latter often published many decades ago. Experience with more targeted approaches, such as rituximab, highlights its efficacy and safety in cases with MCD, and a response rate of around 50% in adults with presumed primary FSGS [37]. Targeted and/or podocyte-directed therapies are likely to replace immunosuppressive measures such as CNIs or cyclophosphamide in the near future.

In unresponsive patients and patients with persistent proteinuria predicting poor prognosis (UPCR >1.5 g/g), priority should be given to nephroprotection through alleviating podocyte injury and loss by controlling systemic and intraglomerular hypertension. As resistant cases with FSGS often have an underlying genetic cause, a genetic analysis should be performed prior to applying specific therapies that might help to reduce proteinuria [68]. More RCT evidence is needed with improved and specific trial design for treating patients with MCD and subclasses of FSGS. The results from multiple ongoing studies are awaited. Hopefully, novel, safe, and effective therapies will soon become available for our patient populations, eventually improving response rates in FSGS and translating into improved prognosis.

## Supplementary Material

gfae025_Supplemental_File

## Data Availability

Not available.
