# Correction to: Management of adult patients with podocytopathies: an update from the ERA Immunonephrology Working Group

**DOI:** 10.1093/ndt/gfae215

**Published:** 2024-10-01

This is a correction to: Safak Mirioglu, Lisa Daniel-Fischer, Ilay Berke, Syed Hasan Ahmad, Ingeborg M Bajema, Annette Bruchfeld, Gema M Fernandez-Juarez, Jürgen Floege, Eleni Frangou, Dimitrios Goumenos, Megan Griffith, Sarah M Moran, Cees van Kooten, Stefanie Steiger, Kate I Stevens, Kultigin Turkmen, Lisa C Willcocks, Andreas Kronbichler, Management of adult patients with podocytopathies: an update from the ERA Immunonephrology Working Group, *Nephrology Dialysis Transplantation*, Volume 39, Issue 4, April 2024, Pages 569–580, https://doi.org/10.1093/ndt/gfae025

In the supplementary material, the phrase:

‘Gonadal toxicity in women was shown in patients exposed to a cumulative dose of greater than 5 grams [17].’

Has been corrected to read:

‘A paucity of data exists related to secondary amenorrhea following cyclophosphamide exposure in podocytopathies. In SLE, exposure to ≥8 g/m2 of cyclophosphamide in patients ≥32 years predicted sustained amenorrhea. Even at 5 g/m2, the estimated risk was 32% (PMID: 12 375 322). In women with AAV (mean age of 35), diminished ovarian reserve was reported in 75% of participants who received oral cyclophosphamide (PMID: 22127969).’

