# Supplementary material for: Management of adult patients with podocytopathies: an update from the ERA Immunonephrology Working Group
Source: Nephrol Dial Transplant. 2024 Feb 10;39(4):569–80. doi: 10.1093/ndt/gfae025 (PMC11024823; doi:10.1093/ndt/gfae025)
Supplement: gfae025_Supplemental_File [file gfae025_Supplemental_File.docx]

**Supplemental File to:**

**Special Report**

**Title: An Update on Management of Adult Patients with Minimal Change Disease and Focal Segmental Glomerulosclerosis – a European Renal Association – Immunonephrology Working Group (ERA-IWG) Initiative**

**Alternative approaches to manage relapsing or resistant FSGS**

Despite the lack of RCTs in adult patients with FSGS, observational data suggested that rituximab was efficient in reducing relapses, and reduction or withdrawal of steroids and CNIs in especially FR/SD disease [1]. In 2014, the NEMO Study Group showed a decline in relapse rates. An analysis of available studies in 2014 concluded that rituximab was successful in reducing relapses and concomitant immunosuppression despite the low number of FSGS patients compared to MCD [2, 3]. However other reports, including the Spanish GLOSEN registry, did not confirm these findings [4, 5]. An observational study from India demonstrated the efficacy in CNI-dependent FSGS, and the Italian national cohort showed that rituximab was beneficial in steroid-dependent but not in steroid-resistant cases. Furthermore, a reassuring safety profile has been demonstrated for rituximab in many studies [6, 7]. Infusion-related reactions are generally mild; and hepatitis B virus reactivation, which has been associated with use of rituximab, can be prevented by screening and appropriate prophylactic measures [8]. Persistent hypogammaglobulinemia with prolonged rituximab use and late-onset neutropenia may nevertheless be of concern. Hypogammaglobulinemia is also quite common in patients with nephrotic syndrome, so it may be difficult to determine the individual effect of nephrotic syndrome and rituximab on hypogammaglobulinemia risk in these patients [1]. Late-onset neutropenia has been reported to be rare in patients with MCD/FSGS treated with rituximab [8, 9]. In conclusion, based on the safety profile, rituximab may be the preferred agent in the treatment of FR/SD and CNI-dependent FSGS. From a financial standpoint, treatment costs have been reduced by the use of biosimilars [10]. No convincing data supporting rituximab use in steroid- and CNI-resistant FSGS have been published so far.

Cyclophosphamide has been shown to be a reliable treatment option for FR/SD MCD with lower relapse rates than CNIs, and its efficacy in FSGS has been demonstrated, as well [11, 12]. It is a cheap and widely available alkylating agent but comes with adverse effects on various organs/systems, such as infertility, bladder toxicity, and oncogenicity [1, 13]. Malignancies associated with cyclophosphamide have been reported to manifest even years after the exposure, therefore, the true risk of neoplasm may have been underestimated [14]. Most of these adverse events have been associated with higher cumulative doses [15, 16]. The increased risk of bladder cancer was shown in patients exceeding 30 grams of cyclophosphamide in total, and doses below 10 grams were not related to an increase in malignancies except squamous cell carcinoma. Gonadal toxicity in women was shown in patients exposed to a cumulative dose of greater than 5 grams [17]. Despite these drawbacks, cyclophosphamide remains an important immunosuppressive agent that induces durable remissions and thus limits exposure to steroids.

Mycophenolic acid analogs have been considered to be useful steroid-sparing agents according to the data of observational studies and small trials [18, 19]. In 2011, a clinical trial evaluated the efficacy of mycophenolate mofetil in combination with dexamethasone compared to cyclosporine in SR FSGS in mainly children and adolescents [20]. Partial remission was reported in 33.3% of patients treated with mycophenolate mofetil and dexamethasone compared to 45.8% of patients with cyclosporine. Although the results looked promising, the trial was quite underpowered because only a quarter of the initial enrollment target was reached [21]. Thus, the KDIGO 2021 GD guideline decided to rescind the class 2C recommendation of mycophenolate mofetil plus dexamethasone which was provided in the 2012 guideline [21, 22]. Small studies suggested a potential role for adrenocorticotropic hormone (ACTH) in the treatment of FSGS [23, 24]. However, ACTH comes with a higher rate of adverse events and higher costs than synthetic oral glucocorticoids [25], and evidence stems from low quality cases series/studies precluding its widespread use.

**Calcineurin inhibitor therapy in FSGS**

CNI therapy in FSGS should be separately evaluated for three indications: initial treatment, FR/SD disease, and SR FSGS. In patients who are intolerant to prolonged courses of high-dose glucocorticoids due to adverse events and those who may have relative contraindications for such therapy (i.e., in case of obesity, psychiatric conditions, uncontrolled diabetes mellitus or severe osteoporosis), CNIs can be chosen as the initial treatment. Observational data have shown efficacy when used alone or in combination with low-dose glucocorticoids [21, 26-28]. CNIs were shown to be equally efficacious as cyclophosphamide in inducing remission in FSGS; however, relapses were more common when the drug was withdrawn, and protracted treatment duration increases the risk of nephrotoxicity [1, 11, 29].

Data are relatively more robust when it comes to the treatment of patients with SR FSGS. A Cochrane systematic review listed 5 studies of cyclosporine including 240 patients with resistant disease, and cyclosporine with or without prednisone was found to be associated with increased remission rates in an analysis of 231 patients [30]. Cyclosporine has been generally used in combination with prednisone but efficacy of monotherapy has also been reported [30]. Evidence for tacrolimus in comparison with steroids or supportive therapy is more limited. Observational data have, however, demonstrated efficacy and a similar mechanism of action as cyclosporine. Both CNIs have thus been suggested for use in SR FSGS [21]. If no improvement occurs after 6 months of CNI use (trough levels in the therapeutic range), the patient is deemed CNI-resistant [21].

Relapse after withdrawal is associated with CNIs, and has been shown in various studies to occur in 50-80% of patients who achieve remission [11, 29, 31, 32]. Each episode of relapse increases the risk of CKD and the need for repeated immunosuppressive treatment [21]. On the other hand, prolonged use of CNIs to avoid relapse is not without risk [1]. To balance these risks, a minimum CNI use of 12 months has been suggested in responsive patients, followed by a slow taper over a course of 6-12 months [21]. Cyclosporine and tacrolimus should be started at 3-5 mg/kg/day and 0.05-0.1 mg/kg/day, respectively, in divided doses with target trough levels of 100-175 ng/ml and 5-10 ng/ml, respectively, or according to local laboratory methods for transplant recipients, considering the risk of nephrotoxicity [21].

**Extracorporeal therapies in the management of FSGS**

The presumed involvement of circulating permeability factors in the pathogenesis of primary FSGS paved the way for various extracorporeal therapies, such as therapeutic plasma exchange (TPE), immunoadsorption (IAS), and low-density lipoprotein-apheresis (LDL-A) [33]. IAS can selectively remove the probable circulating factors from the plasma using high-affinity columns for adsorption [33]. Small studies showed some efficacy of IAS and TPE in patients with primary FSGS, and IAS has benefits over TPE as it obviates the use of additional albumin or fresh-frozen plasma [34, 35]. LDL-A is the main extracorporeal therapeutic method that has been studied in primary FSGS, and has been FDA-approved for the management of pediatric FSGS cases [33]. Even though its exact mechanism of action is still under debate, LDL-A has been shown to induce remission and increase the response to other immunosuppressive agents [33]. However, most of these data came from observational studies primarily conducted in Japan [36]. The American Society for Apheresis gave class 2C recommendations for LDL-A and TPE, but refrained from providing any formal recommendations on IAS use in steroid-resistant primary FSGS [37, 38]. We think that IAS may be used in this indication, as well, as evidence from recurrent FSGS after kidney transplantation seems to be promising [39]. Nevertheless, these therapies should only be applied to selected patients in expert centers.

**KDIGO 2021: Anticoagulation - what is the evidence for MCD/FSGS?**

Increased thromboembolic risk in nephrotic syndrome, including deep venous thrombosis, renal vein thrombosis and pulmonary embolism, is a well-known complication [40], especially within the first 6 months of diagnosis [41]. However, the exact mechanisms that lead to this hypercoagulable state in nephrotic syndrome are still poorly understood. It has been suggested that these complications stem from the imbalance between urinary loss of anticoagulants and increased liver procoagulant synthesis, platelet activation and aggregability, decreased fibrinolytic activity, localized clotting in the kidney [42], as well as volume depletion, diuretic and/or steroid therapy [43].

Membranous nephropathy is associated with the highest risk of thromboembolism, yet it can also occur in MCD and FSGS [44]. The KDIGO 2021 Work Group expanded the evaluation and the recommendations of thromboembolism in nephrotic syndrome, and included a risk-based treatment algorithm in 2021 [21]. The KDIGO 2021 GD guideline suggests prophylactic anticoagulation when the risk of thromboembolism exceeds the patient-specific risks of a serious bleeding event due to anticoagulation (see Supplemental Table 2) [21].

The risk-based treatment algorithm included in the KDIGO 2021 GD guideline has been established for patients with MN, therefore its value for patients with MCD and FSGS is unclear. It is based on albumin levels and the presence of additional risk factors and concomitant diseases and attempts to account for the bleeding risk as well. To date there are no RCTs addressing prophylactic anticoagulation in nephrotic syndrome, therefore evidence for these recommendations is scarce and derived primarily from case studies or retrospective cohort studies [45]. Especially for MCD and FSGS, data are very limited and only few studies are reporting on the outcome of prophylactic anticoagulation in these patients [41, 46, 47]. Unfractionated heparin, low-molecular-weight heparin and vitamin K antagonists, as well as more recently direct oral anticoagulants (off-label) have been suggested in the KDIGO 2021 GD guideline [21]. Phase 1a study data indicate that the levels of free apixaban are consistent with reports in the literature of individuals without nephrotic syndrome [48], but further studies are warranted to establish the role of direct oral anticoagulants. The duration of full-dose anticoagulation is suggested for 6-12 months and/or for the duration of the nephrotic syndrome for thromboembolic events [21], the duration of prophylactic treatment is not specified in the guideline, but should be based on the duration of the nephrotic syndrome with a continuous risk-benefit analysis.

References:

1. Gauckler P, Shin JI, Alberici F*, et al.* Rituximab in adult minimal change disease and focal segmental glomerulosclerosis - What is known and what is still unknown? Autoimmun Rev 2020;19(11):102671

2. Ruggenenti P, Ruggiero B, Cravedi P*, et al.* Rituximab in steroid-dependent or frequently relapsing idiopathic nephrotic syndrome. J Am Soc Nephrol 2014;25(4):850-863

3. Kronbichler A, Kerschbaum J, Fernandez-Fresnedo G*, et al.* Rituximab treatment for relapsing minimal change disease and focal segmental glomerulosclerosis: a systematic review. Am J Nephrol 2014;39(4):322-330

4. Roccatello D, Sciascia S, Rossi D*, et al.* High-Dose Rituximab Ineffective for Focal Segmental Glomerulosclerosis: A Long-Term Observation Study. Am J Nephrol 2017;46(2):108-113

5. Fernandez-Fresnedo G, Segarra A, González E*, et al.* Rituximab treatment of adult patients with steroid-resistant focal segmental glomerulosclerosis. Clin J Am Soc Nephrol 2009;4(8):1317-1323

6. Ramachandran R, Bharati J, Rao I*, et al.* Persistent CD-19 depletion by rituximab is cost-effective in maintaining remission in calcineurin-inhibitor dependent podocytopathy. Nephrology (Carlton) 2019;24(12):1241-1247

7. Tedesco M, Mescia F, Pisani I*, et al.* The Role of Rituximab in Primary Focal Segmental Glomerular Sclerosis of the Adult. Kidney Int Rep 2022;7(8):1878-1886

8. Kronbichler A, Windpessl M, Pieringer H*, et al.* Rituximab for immunologic renal disease: What the nephrologist needs to know. Autoimmun Rev 2017;16(6):633-643

9. Zonozi R, Wallace ZS, Laliberte K*, et al.* Incidence, Clinical Features, and Outcomes of Late-Onset Neutropenia From Rituximab for Autoimmune Disease. Arthritis Rheumatol 2021;73(2):347-354

10. Jang M, Simoens S, Kwon T. Budget Impact Analysis of the Introduction of Rituximab and Trastuzumab Intravenous Biosimilars to EU-5 Markets. BioDrugs 2021;35(1):89-101

11. Ponticelli C, Edefonti A, Ghio L*, et al.* Cyclosporin versus cyclophosphamide for patients with steroid-dependent and frequently relapsing idiopathic nephrotic syndrome: a multicentre randomized controlled trial. Nephrol Dial Transplant 1993;8(12):1326-1332

12. Ren H, Shen P, Li X*, et al.* Tacrolimus versus cyclophosphamide in steroid-dependent or steroid-resistant focal segmental glomerulosclerosis: a randomized controlled trial. Am J Nephrol 2013;37(1):84-90

13. Ponticelli C, Escoli R, Moroni G. Does cyclophosphamide still play a role in glomerular diseases? Autoimmun Rev 2018;17(10):1022-1027

14. Faurschou M, Sorensen IJ, Mellemkjaer L*, et al.* Malignancies in Wegener's granulomatosis: incidence and relation to cyclophosphamide therapy in a cohort of 293 patients. J Rheumatol 2008;35(1):100-105

15. Monach PA, Arnold LM, Merkel PA. Incidence and prevention of bladder toxicity from cyclophosphamide in the treatment of rheumatic diseases: a data-driven review. Arthritis Rheum 2010;62(1):9-21

16. Heijl C, Westman K, Höglund P*, et al.* Malignancies in Patients with Antineutrophil Cytoplasmic Antibody-associated Vasculitis: A Population-based Cohort Study. J Rheumatol 2020;47(8):1229-1237

17. Luong SN, Isaacs A, Liu Z*, et al.* A systematic review and meta-analysis of the gonadotoxic effects of cyclophosphamide and benefits of gonadotropin releasing hormone agonists (GnRHa) in women of child-bearing age with autoimmune rheumatic disease. Expert Rev Clin Immunol 2020;16(3):321-333

18. Day CJ, Cockwell P, Lipkin GW*, et al.* Mycophenolate mofetil in the treatment of resistant idiopathic nephrotic syndrome. Nephrol Dial Transplant 2002;17(11):2011-2013

19. Senthil Nayagam L, Ganguli A, Rathi M*, et al.* Mycophenolate mofetil or standard therapy for membranous nephropathy and focal segmental glomerulosclerosis: a pilot study. Nephrol Dial Transplant 2008;23(6):1926-1930

20. Gipson DS, Trachtman H, Kaskel FJ*, et al.* Clinical trial of focal segmental glomerulosclerosis in children and young adults. Kidney Int 2011;80(8):868-878

21. KDIGO 2021 Clinical Practice Guideline for the Management of Glomerular Diseases. Kidney Int 2021;100(4S):S1-S276

22. Chapter 6: Idiopathic focal segmental glomerulosclerosis in adults. Kidney Int Suppl (2011) 2012;2(2):181-185

23. Hogan J, Bomback AS, Mehta K*, et al.* Treatment of idiopathic FSGS with adrenocorticotropic hormone gel. Clin J Am Soc Nephrol 2013;8(12):2072-2081

24. Kittanamongkolchai W, Cheungpasitporn W, Zand L. Efficacy and safety of adrenocorticotropic hormone treatment in glomerular diseases: a systematic review and meta-analysis. Clin Kidney J 2016;9(3):387-396

25. Chakraborty R, Mehta A, Nair N*, et al.* ACTH Treatment for Management of Nephrotic Syndrome: A Systematic Review and Reappraisal. Int J Nephrol 2020;2020:2597079

26. Goumenos DS, Tsagalis G, El Nahas AM*, et al.* Immunosuppressive treatment of idiopathic focal segmental glomerulosclerosis: a five-year follow-up study. Nephron Clin Pract 2006;104(2):c75-82

27. Duncan N, Dhaygude A, Owen J*, et al.* Treatment of focal and segmental glomerulosclerosis in adults with tacrolimus monotherapy. Nephrol Dial Transplant 2004;19(12):3062-3067

28. Chávez-Mendoza CA, Niño-Cruz JA, Correa-Rotter R*, et al.* Calcineurin Inhibitors With Reduced-Dose Steroids as First-Line Therapy for Focal Segmental Glomerulosclerosis. Kidney Int Rep 2019;4(1):40-47

29. Niaudet P, Habib R. Cyclosporine in the treatment of idiopathic nephrosis. J Am Soc Nephrol 1994;5(4):1049-1056

30. Hodson EM, Sinha A, Cooper TE. Interventions for focal segmental glomerulosclerosis in adults. Cochrane Database Syst Rev 2022;2(2):CD003233

31. Cattran DC, Appel GB, Hebert LA*, et al.* A randomized trial of cyclosporine in patients with steroid-resistant focal segmental glomerulosclerosis. North America Nephrotic Syndrome Study Group. Kidney Int 1999;56(6):2220-2226

32. Fervenza FC, Appel GB, Barbour SJ*, et al.* Rituximab or Cyclosporine in the Treatment of Membranous Nephropathy. N Engl J Med 2019;381(1):36-46

33. Raina R, Wang J, Sharma A*, et al.* Extracorporeal Therapies in the Treatment of Focal Segmental Glomerulosclerosis. Blood Purif 2020;49(5):513-523

34. Haas M, Godfrin Y, Oberbauer R*, et al.* Plasma immunadsorption treatment in patients with primary focal and segmental glomerulosclerosis. Nephrol Dial Transplant 1998;13(8):2013-2016

35. Franke D, Zimmering M, Wolfish N*, et al.* Treatment of FSGS with plasma exchange and immunadsorption. Pediatr Nephrol 2000;14(10-11):965-969

36. Kronbichler A, Gauckler P, Lee KH*, et al.* Immunoadsorption in nephrotic syndrome: Where are we now and where are we going from here? Atheroscler Suppl 2019;40:55-60

37. Cervantes CE, Bloch EM, Sperati CJ. Therapeutic Plasma Exchange: Core Curriculum 2023. Am J Kidney Dis 2023;81(4):475-492

38. Connelly-Smith L, Alquist CR, Aqui NA*, et al.* Guidelines on the Use of Therapeutic Apheresis in Clinical Practice - Evidence-Based Approach from the Writing Committee of the American Society for Apheresis: The Ninth Special Issue. J Clin Apher 2023;38(2):77-278

39. Allard L, Kwon T, Krid S*, et al.* Treatment by immunoadsorption for recurrent focal segmental glomerulosclerosis after paediatric kidney transplantation: a multicentre French cohort. Nephrol Dial Transplant 2018;33(6):954-963

40. Gordon-Cappitelli J, Choi MJ. Prophylactic Anticoagulation in Adult Patients with Nephrotic Syndrome. Clin J Am Soc Nephrol 2020;15(1):123-125

41. Mahmoodi BK, ten Kate MK, Waanders F*, et al.* High absolute risks and predictors of venous and arterial thromboembolic events in patients with nephrotic syndrome: results from a large retrospective cohort study. Circulation 2008;117(2):224-230

42. Singhal R, Brimble KS. Thromboembolic complications in the nephrotic syndrome: pathophysiology and clinical management. Thromb Res 2006;118(3):397-407

43. Glassock RJ. Prophylactic anticoagulation in nephrotic syndrome: a clinical conundrum. J Am Soc Nephrol 2007;18(8):2221-2225

44. Kerlin BA, Ayoob R, Smoyer WE. Epidemiology and pathophysiology of nephrotic syndrome-associated thromboembolic disease. Clin J Am Soc Nephrol 2012;7(3):513-520

45. Lin R, McDonald G, Jolly T*, et al.* A Systematic Review of Prophylactic Anticoagulation in Nephrotic Syndrome. Kidney Int Rep 2020;5(4):435-447

46. Kelddal S, Nykjær KM, Gregersen JW*, et al.* Prophylactic anticoagulation in nephrotic syndrome prevents thromboembolic complications. BMC Nephrol 2019;20(1):139

47. Rostoker G, Durand-Zaleski I, Petit-Phar M*, et al.* Prevention of thrombotic complications of the nephrotic syndrome by the low-molecular-weight heparin enoxaparin. Nephron 1995;69(1):20-28

48. Derebail VK, Zhu J, Crawford ML*, et al.* Pharmacokinetics and Pharmacodynamics of Apixaban in Nephrotic Syndrome: Findings From a Phase 1a Trial. Am J Kidney Dis 2023;81(3):373-376

49. Chapter 5: Minimal-change disease in adults. Kidney Int Suppl (2011) 2012;2(2):177-180

**Supplemental Table 1.** Comparison of the most important recommendations and practice points of the 2021 KDIGO Clinical Practice Guideline for the Management of Glomerular Diseases with the 2012 guideline [21, 22, 49].

| **Topic** | **2012 KDIGO Guideline** | **2021 KDIGO Guideline** |
| --- | --- | --- |
| **Minimal change disease (MCD)** | | |
| Initial treatment of adults | R 5.1.1. The group recommends GCs to be given for initial treatment of nephrotic syndrome.  R 5.1.5. For patients with relative contraindications or intolerance to high-dose GCs (e.g., uncontrolled diabetes, psychiatric conditions, severe osteoporosis), the group suggests oral CYC or CNIs as discussed in FR MCD. | R 5.3.1. The group recommends high dose oral GCs for initial treatment of MCD.  PP 5.3.1: Algorithm for the initial treatment of MCD in adults:   \| **Situation** \| **Suggestion** \| \| --- \| --- \| \| No contraindications for GCs \| GCs \| \| Contraindications for GCs \| CYC  CNI  MMF/MPS and reduced dose GCs  RTX? \|   PP 5.3.3: Begin tapering of GCs 2 weeks after complete remission.  PP 5.3.5: For patients in whom glucocorticoids may be relatively contraindicated, consider initial therapy with CYC, a CNI, or MMF. |
| Infrequent relapses | R 5.1.6. The group suggests using the same initial dose and duration of corticosteroids for infrequent relapses as in recommendations 5.1.2, 5.1.3, and 5.1.4. | PP 5.3.1.2: Treat infrequent relapses with GCs. |
| FR/SD disease | R 5.2.1: The group suggests oral CYC 2-2.5 mg/kg/d for 8 weeks.  R 5.2.2: The group suggests CNIs (CsA 3-5 mg/kg/d or Tac 0.05-0.1 mg/kg/d in divided doses) for 1-2 years for FR/SD MCD patients who have relapsed despite CYC, or for patients who wish to preserve their fertility.  R 5.2.3: The group suggests MMF 500-1000 mg twice daily for 1-2 years for patients who are intolerant of GCs, CYC, and CNIs. | R 5.3.1.1: The group recommends CYC, RTX, CNIs, or mycophenolic acid analogs for the treatment of FR/SD MCD, rather than prednisone alone or no treatment.  PP 5.3.1.1: Algorithm for treatment of FR/SD MCD in adults:   \| **Situation** \| **Suggestion** \| \| --- \| --- \| \| No previous CYC  No patient preference \| CYC \| \| Previous CYC  Patient wishing to avoid CYC \| RTX  CNI  MMF/MPS \| |
| **Focal segmental glomerulosclerosis (FSGS)** | | |
| Evaluation | R 6.1.1: Undertake thorough evaluation to exclude secondary forms of FSGS.  R 6.1.2: Do not routinely perform genetic testing. | PP 6.1.1.1: Adults with FSGS who do not have nephrotic syndrome should be evaluated for a secondary cause.  PP 6.1.2.1: Genetic testing may be beneficial for selected patients with FSGS who should be referred to specialized centers with such expertise. |
| Initial treatment | R 6.2.1: The group recommends GC and immunosuppressive therapy to be considered only in idiopathic FSGS associated with clinical features of the nephrotic syndrome.  R 6.2.5: The group suggests CNIs to be considered as first-line therapy for patients with relative contraindications or intolerance to high-dose GCs (e.g., uncontrolled diabetes, psychiatric conditions, severe osteoporosis). | PP 6.2.1.1: Immunosuppression should not be used in adults with FSGS of undetermined cause, or in those with secondary FSGS.  R 6.2.2.1: The group recommends that high-dose oral GCs be used as the first-line immunosuppressive treatment for primary FSGS.  PP 6.2.2.4: In adults with relative contraindications or intolerance to GCs, alternative immunosuppression with CNIs should be considered as the initial therapy in patients with primary FSGS. |
| Relapse | R 6.3.1: The group suggests that a relapse of nephrotic syndrome is treated as per the recommendations for relapsing MCD in adults. | PP 6.3.5.1: Adults with previous steroid-sensitive primary FSGS who experience a relapse can be treated using the same approach as that for adults with relapsing MCD. |
| Steroid-resistant disease | R 6.4.1: For steroid-resistant FSGS, the group suggests that CsA at 3-5 mg/kg/d in divided doses be given for at least 4–6 months.  R 6.4.2: If there is a partial or complete remission, the group suggests continuing CsA treatment for at least 12 months, followed by a slow taper.  R 6.4.3: The group suggests patients with steroid-resistant FSGS, who do not tolerate cyclosporine, to be treated with a combination of MMF and high-dose dexamethasone. | R 6.3.1.1: For adults with steroid-resistant primary FSGS, we recommend that CsA or Tac be given for 6 months rather than continuing with GC monotherapy or not treating.  PP 6.3.3.1: Adults with steroid-resistant primary FSGS who respond to CNI treatment should receive CNIs for a minimum of 12 months to minimize the risk of relapses.  PP 6.3.4.1: Adults who have steroid-resistant primary FSGS with resistance to or intolerance of CNIs should be referred to specialized centers for consideration of re-biopsy, alternative treatment, or enrollment in a clinical trial. |

**Abbreviations:** CNI: calcineurin inhibitor, CsA: cyclosporine, CYC: cyclophosphamide, FR: frequently relapsing, GC: glucocorticoid, MCD: minimal change disease, MMF: mycophenolate mofetil, MPS: mycophenolate sodium, FSGS: focal segmental glomerulosclerosis, KDIGO: Kidney Disease: Improving Global Outcomes, PP: practice point, R: recommendation, RTX: rituximab, SD: steroid-dependent, Tac: tacrolimus.

**Supplemental Table 2.** Weighing the benefits and risks of anticoagulation in patients with nephrotic syndrome. Specific risk factors providing a rationale to use anticoagulation, as well as relative or absolute contraindications as highlighted in KDIGO 2021 glomerular diseases guideline. It is mentioned that especially patients with membranous nephropathy carry a particularly high risk of thromboembolic events.

| For thromboembolic events, full-dose anticoagulation is required for 6-12 months and/or for the duration of the nephrotic syndrome | Prophylactic full-dose anticoagulation should be considered if: | Relative or absolute contraindications to prophylactic anticoagulation |
| --- | --- | --- |
| Venous thrombosis  Arterial thrombosis  Pulmonary embolus  Nonvalvular atrial fibrillation | Serum albumin < 20-25 g/l (< 20, if bromocresol purple is used; < 25, if bromocresol green is used) and any of the following:  - Proteinuria > 10 g/d  - Body mass index > 35 kg/m2  - Genetic disposition for thromboembolism  - Heart failure New York Heart Association class III or IV  - Recent orthopedic or abdominal surgery  - Prolonged immobilization | - Patient preference/ability to adhere  - Bleeding diathesis  - Central nervous system lesion prone to hemorrhage  - Genetic mutations influencing warfarin metabolism/efficacy  - Frailty (falls)  - Prior gastrointestinal bleed |
